# Palmitoylation of Hepatitis C Virus NS2 Regulates Its Subcellular Localization and NS2-NS3 Autocleavage

**DOI:** 10.1128/JVI.00906-19

**Published:** 2019-12-12

**Authors:** Ming-Jhan Wu, Saravanabalaji Shanmugam, Christoph Welsch, MinKyung Yi

**Affiliations:** a Department of Microbiology and Immunology, University of Texas Medical Branch at Galveston, Galveston, Texas, USA; b Department of Internal Medicine I, Goethe University Hospital, Frankfurt/Main, Germany; University of Southern California

**Keywords:** DRM, NS2, autoprocessing, hepatitis C virus, palmitoylation

## Abstract

Hepatitis C virus (HCV) nonstructural protein 2 (NS2) is a multifunctional protein implicated in both HCV RNA replication and virus particle assembly. NS2-encoded cysteine protease is responsible for autoprocessing of NS2-NS3 precursor, an essential step in HCV RNA replication. NS2 also promotes HCV particle assembly by recruiting envelope protein 2 (E2) to the virus assembly sites located at the detergent-resistant membranes (DRM). However, the fundamental mechanism regulating multiple functions of NS2 remains unclear. In this study, we discovered that NS2 is palmitoylated at the position 113 cysteine residue (NS2/C113) when expressed by itself in cells and during infectious-HCV replication. Blocking NS2 palmitoylation by introducing an NS2/C113S mutation reduced NS2-NS3 autoprocessing and impaired HCV RNA replication. Replication of the NS2/C113S mutant was restored by inserting an encephalomyocarditis virus (EMCV) internal ribosome entry site (IRES) between NS2 and NS3 to separate the two proteins independently of NS2-mediated autoprocessing. These results suggest that NS2 palmitoylation is critical for HCV RNA replication by promoting NS2-NS3 autoprocessing. The NS2/C113S mutation also impaired infectious-HCV assembly, DRM localization of NS2 and E2, and colocalization of NS2 with Core and endoplasmic reticulum lipid raft-associated protein 2 (Erlin-2). In conclusion, our study revealed that two major functions of NS2 involved in HCV RNA replication and virus assembly, i.e., NS2-NS3 autoprocessing and E2 recruitment to the DRM, are regulated by palmitoylation at NS2/C113. Since S-palmitoylation is reversible, NS2 palmitoylation likely allows NS2 to fine tune both HCV RNA replication and infectious-particle assembly.

**IMPORTANCE** Chronic infection with hepatitis C virus (HCV) is a major cause of severe liver diseases responsible for nearly 400,000 deaths per year. HCV NS2 protein is a multifunctional regulator of HCV replication involved in both viral-genome replication and infectious-virus assembly. However, the underlying mechanism that enables the protein to participate in multiple steps of HCV replication remains unknown. In this study, we discovered that NS2 palmitoylation is the master regulator of its multiple functions, including NS2-mediated self-cleavage and HCV envelope protein recruitment to the virus assembly sites, which in turn promote HCV RNA replication and infectious-particle assembly, respectively. This newly revealed information suggests that NS2 palmitoylation could serve as a promising target to inhibit both HCV RNA replication and virus assembly, representing a new avenue for host-targeting strategies against HCV infection.

## Abstract (precis)

Chronic infection with hepatitis C virus (HCV) is a major cause of severe liver diseases responsible for nearly 400,000 deaths per year. HCV NS2 protein is a multifunctional regulator of HCV replication involved in both viral-genome replication and infectious-virus assembly. However, the underlying mechanism that enables the protein to participate in multiple steps of HCV replication remains unknown. In this study, we discovered that NS2 palmitoylation is the master regulator of its multiple functions, including NS2-mediated self-cleavage and HCV envelope protein recruitment to the virus assembly sites, which in turn promote HCV RNA replication and infectious-particle assembly, respectively. This newly revealed information suggests that NS2 palmitoylation could serve as a promising target to inhibit both HCV RNA replication and virus assembly, representing a new avenue for host-targeting strategies against HCV infection.

## INTRODUCTION

Hepatitis C virus (HCV) is an enveloped, positive-sense RNA virus belonging to the genus *Hepacivirus* in the family *Flaviviridae*. HCV is a major causative agent associated with severe liver diseases, including chronic hepatitis, cirrhosis, and hepatocellular carcinoma in the human population (1). Despite the availability of direct-acting antivirals (DAAs) that could cure ∼95% of HCV-infected individuals (2), HCV prevalence, affecting more than 71 million people, is not decreasing in a meaningful way due to the limited uptake of DAA treatments and ongoing new infections, especially for those in high-risk groups (3, 4).

While HCV is still the sole member of the hepaciviruses that infects humans, multiple other hepaciviruses were discovered recently from diverse host ranges, including horses, cattle, rodents, bats, New and Old World primates, and even a shark (see reference 5 for a detailed review). Since there is no robust animal model for HCV infection to help us better understand virus-host interaction, some of these hepaciviruses have been considered for surrogate animal models of HCV infection (6). A recent report by Hartlage et al. demonstrated the usefulness of a rodent hepacivirus (RHV) model to define hepacivirus immunity and to evaluate the efficacy of prophylactic vaccination (7).

HCV has a single open reading frame (ORF) that is processed by the viral and host cellular proteases. Host signal peptidase is responsible for the processing of N-terminally encoded Core, E1, E2, p7, and NS2 (8, 9). A C-terminally encoded nonstructural polyprotein (NS3-NS4A-NS4B-NS5A-NS5B) is processed by NS3 serine protease and its cofactor, NS4A (10). Processing between NS2 and NS3 is mediated by the NS2 cysteine protease catalytic domain and regulated by the NS3 N-terminal domain (11–13). NS2 is dispensable for HCV RNA replication (14). However, inhibiting NS2-NS3 autoprocessing by mutating the catalytic residues in NS2 was shown to impair HCV replication by reducing the availability of NS3 (15).

HCV NS2 is a transmembrane protein consisting of three N-terminal transmembrane domains and a C-terminal domain encoding a cysteine protease catalytic subunit (11, 16, 17). NS2 forms a dimeric protease with two composite active sites in which one monomer contributes histidine and glutamate residues and the other contributes the cysteine residue (11, 12). NS2-NS3 autoprocessing is the only cleavage event mediated by the NS2 protease. A recent report by Boukadida et al. demonstrated that NS2 proteins from other hepaciviruses, including those from equine, bat, rodent, and New and Old World primate viruses, are also a dimeric cysteine proteases formed by composite active sites and exhibit intrinsic proteolytic activity, similar to HCV NS2 (12). Considering the divergent NS2 sequences among different hepaciviruses, this unique mode of autoprocessing commonly shared by different NS2s indicates the functional significance of NS2-NS3 autocleavage in the hepacivirus life cycle (12).

HCV NS2 also plays a critical role in HCV particle assembly. We and others have shown that NS2 promotes HCV assembly by recruiting HCV envelope proteins to the virus assembly sites via interactions with both structural (E1 and E2) and nonstructural (NS3 and NS5A) proteins (17–20). Recently, we reported that NS2 is localized to the detergent-resistant membranes (DRM) in a p7-dependent manner and that inhibiting NS2 localization to the DRM also inhibited E2 localization to the DRM and infectious-virus assembly (21). The DRM within the endoplasmic reticulum (ER-DRM) is likely an HCV particle assembly site, as HCV assembly was shown to occur in the ER (22, 23), and not only was a significant portion of HCV Core detected in the DRM fractions, but also the localization of Core, NS2, and E2 to the DRM, as well as infectious-HCV production, was inhibited by short-term treatment with the cholesterol-extracting agent methyl-β-cyclodextrin (MβCD) (21).

As a transmembrane protein, it has been unclear how NS2 could associate with the DRM. Recently, Levental et al. demonstrated that lipid raft (DRM) affinity for the majority of integral membrane proteins depended on their palmitoylation by the membrane-embedded palmitoyl acyltransferase (PAT) (24). Therefore, the goal of this investigation was to assess whether NS2 could be modified by palmitoylation and, if so, whether NS2 palmitoylation might regulate its DRM localization. Our data demonstrated that HCV NS2 is palmitoylated during HCV replication and that preventing NS2 palmitoylation inhibited not only NS2 and E2 localization to the DRM, but also virus particle assembly. In addition, our data indicated that NS2 palmitoylation enhances NS2-NS3 autoprocessing, promoting HCV RNA replication. These results indicate that NS2 palmitoylation regulates dual functional roles of NS2 in both RNA replication and virus particle assembly by controlling both NS2 autoprocessing and its localization to the virus assembly sites at the DRM.

## RESULTS

### Palmitoylation at NS2 residue C113 regulated its subcellular localization in an ectopic expression system.

**FIG 1:**
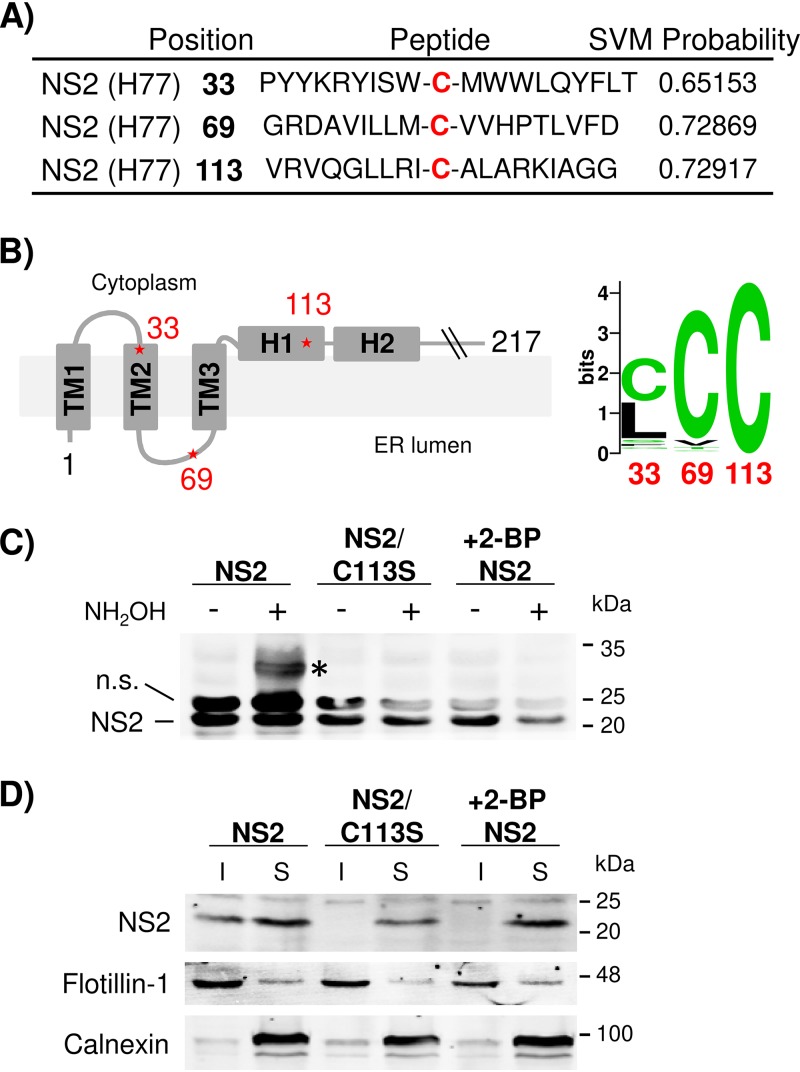
HCV NS2 palmitoylation at the C113 residue promotes its localization to the detergent-insoluble fraction. (A) Palmitoylation probabilities of different cysteine residues in gt1a HCV NS2 analyzed using WAP-Palm software. The support vector machine (SVM) probabilities with low and high thresholds were 0.6 and 0.8, respectively. (B) (Left) Predicted membrane topology model of the NS2 N-terminal domain. The asterisks indicate the putative locations of three potential palmitoylation sites at NS2 residues 33, 69, and 113. (Right) Sequence logo depiction of conservation of NS2 residues 33, 69, and 113 among 672 HCV sequences derived from all the genotypes deposited in the Los Alamos HCV database. (C) 293T cell lysates expressing NS2 or the NS2/C113S mutant were subjected to an APEGS reaction in the presence or absence of NH_2_OH (1 M) in a reaction mixture containing NEM (10 mM) and mPEG-Mal (1 mM) to detect palmitoylated protein. The PEGylated protein is indicated by an asterisk. The cells were treated with 2-bromopalmitate (100 μM) for 16 h posttransfection. n.s., nonspecific bands. (D) 293T cells expressed NS2 and NS2/C113S in gt1a H77 treated with 100 μM 2-BP for 16 h followed by cold 1% TX-100 cell lysate separated into detergent-soluble (S) and -insoluble (I) fractions by low-speed centrifugation (500 × *g*; 5 min). (C and D) The data are representative of three independent experiments.

The potential palmitoylation site(s) in HCV NS2 was analyzed using the WAP-Palm algorithm described by Shi et al. (25), which was developed to predict protein palmitoylation sites by combining multiple features, including the amino acid composition, sequence position information, physicochemical properties, and evolutionary information. This analysis predicted a high probability of palmitoylation at genotype 1a H77 NS2 residues C33, C69, and C113 (Fig. 1A). A nuclear magnetic resonance (NMR)-based structural model for NS2 N-terminal transmembrane (TM) domains reported by Jirasko et al. (17) predicted that C33 and C69 are located at the cytoplasmic side of TM2 and the ER luminal domain linking TM2 and TM3, respectively (Fig. 1B, left). The crystal structure of the C-terminal domain of NS2 reported by Lorenz et al. placed C113 at the membrane-facing helix 1 (H1) immediately following TM3 (Fig. 1B, left). The sequence analysis indicated that C33 is highly variable among different HCV genotypes (Fig. 1B, right). NS2 palmitoylation at C69 is less likely, since secreted proteins were the main target of protein palmitoylation in the ER lumen (26, 27). Therefore, NS2/C113, which showed complete conservation between different HCV genotypes (based on analysis of 672 HCV sequences deposited in the Los Alamos HCV database), was chosen for further analysis for its palmitoylation potential (Fig. 1B, right). We measured the NS2 palmitoylation by using a methoxy-polyethylene glycol–maleimide (mPEG-mal)-based procedure called acyl-PEGyl exchange gel shift (APEGS), which was shown to specifically label palmitoylated proteins in cells (28). In this procedure, free thiol groups of proteins were preblocked by *N*-ethylmaleimide (NEM), followed by specific cleavage of palmitoyl-cysteine thioester linkage with the hydroxylamine (NH_2_OH). Then, these newly exposed cysteine thiols were labeled with mPEG-mal, which led to a mobility shift of palmitoylated (now PEGylated) protein on SDS-polyacrylamide gel electrophoresis (SDS-PAGE) (see Materials and Methods for details). In brief, wild-type (wt) NS2 and a mutant NS2/C113S encoding a cysteine-to-serine mutation at NS2 residue 113 to prevent potential palmitoylation at the residue were ectopically expressed in HEK293T cells, and an APEGS assay was performed. As shown in Fig. 1C, an extra band detectable by anti-NS2 antibody, migrating at ∼32 kDa, which is ∼10 kDa higher than the expected molecular weight of NS2 at ∼22 kDa, was detected from NH_2_OH-treated cell lysates. Although we used 5 kDa mPEG-mal tag in the APEGS reaction, an ∼10-kDa NS2 mass shift by conjugation of this tag likely represents a single palmitoylation event, as each mPEG-mal conjugation was shown to cause a protein mass shift corresponding to twice the mass of the mPEG-mal tag when analyzed by SDS-PAGE (e.g., a 10-kDa mass shift by 5 kDa mPEG-mal, as described in a study by Percher et al. [29]). The NS2/C113S mutation inhibited ∼32-kDa NS2 band formation, indicating that the NS2/C113 residue is the target of palmitoylation. To further verify the NS2 palmitoylation, we performed the APEGS assay using NS2-expressing cell lysates collected following treatment with the palmitoylation inhibitor 2-bromopalmitate (2-BP). As shown in Fig. 1C, 100 μM 2-BP treatment for 16 h inhibited the formation of an ∼32-kDa NS2 band, confirming that NS2 palmitoylation was responsible for its mass shift in the APGES assay, as expected.

Next, we determined the effect of the palmitoylation-abrogating C113S mutation or treatment with 100 μM 2-BP on NS2 fractionation into detergent-insoluble (I) and -soluble (S) fractions, respectively, following treatment of HEK293T cell lysates expressing NS2 with cold 1% Triton X-100, as described previously (21, 30). Following fractionation, flotillin-1 (a marker of DRM) was mainly detected in the detergent-insoluble fractions (DIF), and calnexin (a non-DRM marker) was mainly fractionated to detergent-soluble fractions (DSF), as shown in Fig. 1D. Under these conditions, we detected a portion of NS2 localized in the DIF, as described previously (21). However, NS2 localization to the DIF was inhibited by either NS2/C113S mutation or treatment with 2-BP (Fig. 1D). In aggregate, these results indicate that NS2/C113 is a target of palmitoylation and that the palmitoylation at this residue could alter NS2 subcellular localization.

### Inhibiting NS2 palmitoylation impaired HCV replication by inhibiting the autoprocessing of NS2-NS3 precursor.

**FIG 2:**
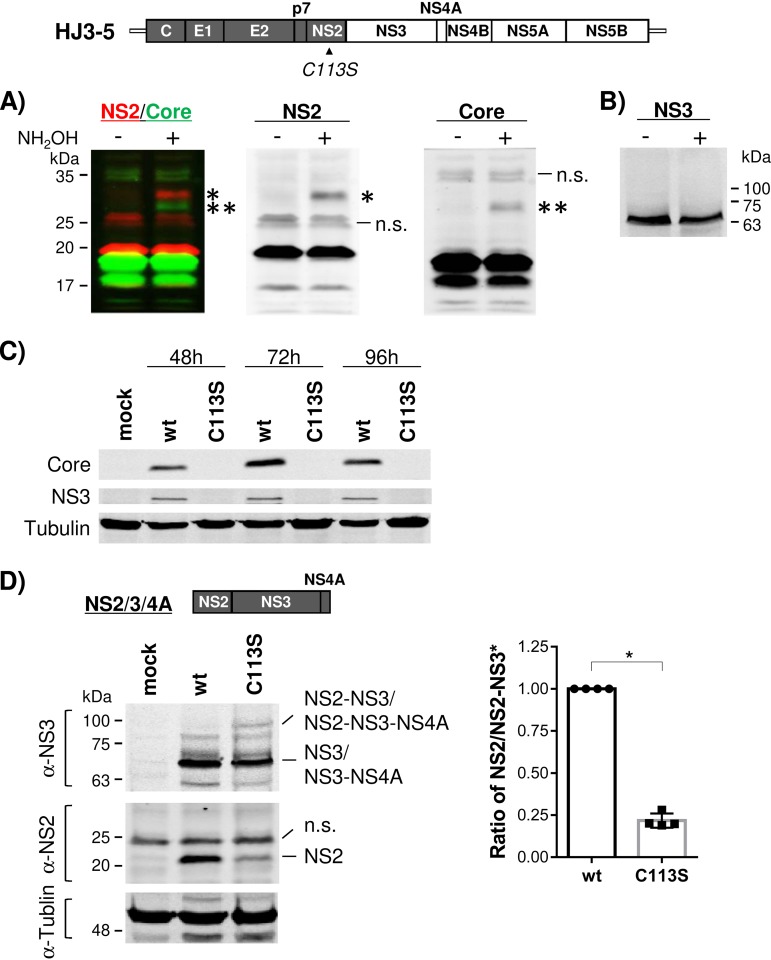
The NS2/C113S mutation inhibited HCV replication and NS2-NS3 autoprocessing. (Top) Organization of infectious HCV (HJ3-5) encoding the Core-to-NS2 sequence from gt1a H77 (shaded) and the rest of the coding sequence from gt2a JFH1 (open). Shown are Western blot results following APEGS assay in the presence and absence of NH_2_OH to detect the potential palmitoylation of NS2 and Core (A) and NS3 (B) by using cell lysates harvested at 48 h postelectroporation of HJ3-5 RNA. (A) (Left) Overlaid multiplex images of NS2 and Core Western blots detected using the Odyssey infrared imaging system. (Middle and right) Separate images for NS2 and Core Western blots, respectively. The NS2 (*) and Core (**) PEGylated proteins are indicated. n.s., nonspecific bands. (C) Western blot of HCV Core and NS3, along with tubulin as a protein-loading control, from cell lysates harvested at different time points postelectroporation of wt or NS2/C113S mutated HJ3-5 RNA. (D) (Left) Western blot of NS2 and NS3 from HEK293T cell lysates harvested at 24 h posttransfection of wt and NS2/C113S mutated p^Flag^NS2-NS3-NS4A plasmid DNA. (Right) Ratios of NS2 to NS2-3 precursor (*) from four independent experiments (mean values with standard deviations). The asterisk indicates a statistically significant difference between paired values based on the Mann-Whitney test (*, *P* < 0.05).

We assessed the status of NS2 palmitoylation during HCV replication to understand the biological significance of the phenomenon. We electroporated infectious-HCV RNA (HJ3-5) (31), containing Core-NS2 sequence from HCV genotype 1a (gt1a) H77 virus within the background of gt2a JFH1 virus, to clonal Huh-7 cells (FT3-7) (32) and then performed the APEGS assay to detect NS2 palmitoylation. As shown in Fig. 2A, we detected a slow-moving ∼32-kDa NS2 protein band, representing PEG-conjugated NS2, in a Western blot analysis following hydroxylamine treatment. These results indicate that NS2 is palmitoylated during HCV replication. We also detected low-level palmitoylation of HCV core protein during HCV replication, which is consistent with previous reports (33, 34) (Fig. 2A). A lack of slow-moving NS3 bands under the same experimental conditions indicated the specificity of the APEGS assay in detecting Core and NS2 palmitoylation (Fig. 2A and B).

Next, we introduced an NS2/C113S mutation into HJ3-5 to determine the functional role of NS2/C113 residue palmitoylation during HCV replication. Surprisingly, we were unable to detect the expression of viral proteins, including Core and NS3, for up to 4 days following electroporation of HJ3-5/C113S mutant RNA into FT3-7 cells, indicating that the NS2/C113S mutation impaired viral replication (Fig. 2C). These data were puzzling, since NS2 was shown to be dispensable for HCV RNA replication (14). As impaired NS2-NS3 processing was shown to impair HCV RNA replication (15), we assessed the effect of NS2/C113S mutation in autoprocessing of NS2-NS3 precursor after expressing wt or NS2/C113S mutant versions of NS2/3/4A polyproteins in HEK293T cells. As shown in Fig. 2D, the level of NS2-NS3 precursor relative to the processed NS2 in the NS2/C113S mutant was higher than that in the wt, indicating that the C113S mutation inhibited NS2-NS3 processing (35). These results suggest that NS2 palmitoylation plays a critical role in HCV replication by promoting NS2-NS3 autoprocessing (see Discussion for details).

### NS2/C113 residue palmitoylation enhanced HCV assembly.

**FIG 3:**
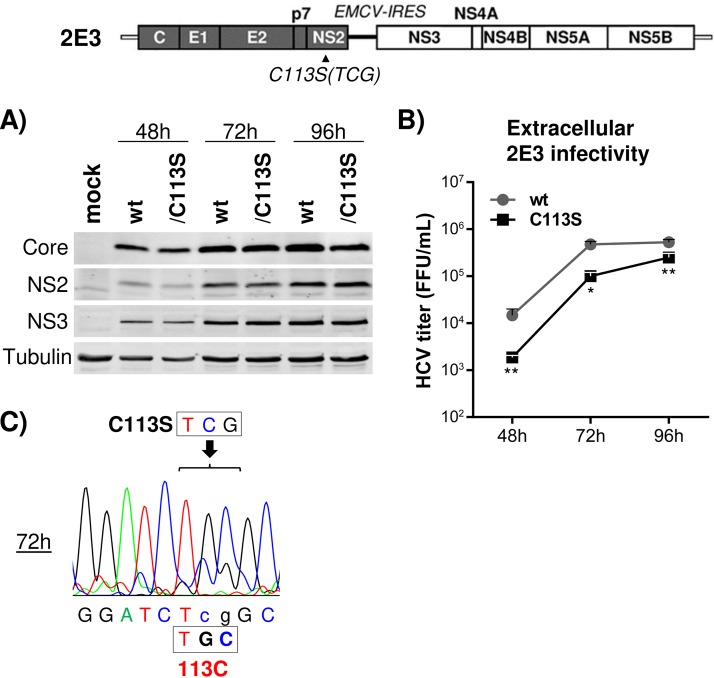
Separation of NS2 and NS3 by inserting the EMCV IRES rescued replication of the C113S mutant. (Top) Organization of 2E3, which encodes the EMCV IRES between NS2 and NS3 in HJ3-5. (A) Western blot of HCV Core, NS2, and NS3, along with tubulin as a protein-loading control, from cell lysates harvested at different time points postelectroporation of wt or NS2/C113S mutant RNA. (B) Extracellular HCV titer determined by using culture supernatants bathing cells harvested at different time points postelectroporation of wt or NS2/C113S mutated 2E3 RNA. The graph shows means with standard deviations from three independent experiments with two replicates. The asterisks indicate statistically significant differences between paired values based on the Mann-Whitney test: **, *P* < 0.005; *, *P* < 0.05. (C) Sequencing chromatogram showing partial reversion of the NS2/C113S mutation to wt detected from cell lysates at 72 h postelectroporation of NS2/C113S mutated 2E3 RNA.

We generated an HCV derivative named 2E3, which encodes the encephalomyocarditis virus (EMCV) internal ribosome entry site (IRES) between the NS2 and NS3 coding regions in HJ3-5, effectively eliminating any NS2-NS3-processing-related problems affecting HCV RNA replication. As shown in Fig. 3A, HJ3-5/2E3 and its NS2/C113S mutant showed comparable courses of viral protein accumulation up to 96 h of culture following electroporation of the respective RNAs into FT3-7 cells. The rescue of an NS2/C113S mutation-mediated defect of HCV replication by EMCV IRES-mediated separation of NS2 and NS3 indicates that the C113S mutation has minimal effect on HCV RNA replication *per se* and supports our assessment that impaired replication of HJ3-5 by the NS2/C113S mutation (Fig. 2C) was due to impaired NS2-NS3 processing. On the other hand, we detected about 7-, 5-, and 2-fold lower extracellular viral titers from the NS2/C113S mutant than wt 2E3 at 48, 72, and 96 h postelectroporation, respectively (Fig. 3B). Sequencing of NS2/C113S mutant RNA at the 72-h time point revealed that the majority of the NS2/C113S (TCG codon) mutation had reverted to the wt cysteine (TGC codon) sequence, providing an explanation for the lower viral production inhibition by the NS2/C113S mutation at later time points of viral replication periods (Fig. 3B and C). The reversion of the NS2/C113S mutation to the wt sequence suggests the critical advantage of NS2 palmitoylation in HCV propagation.

**FIG 4:**
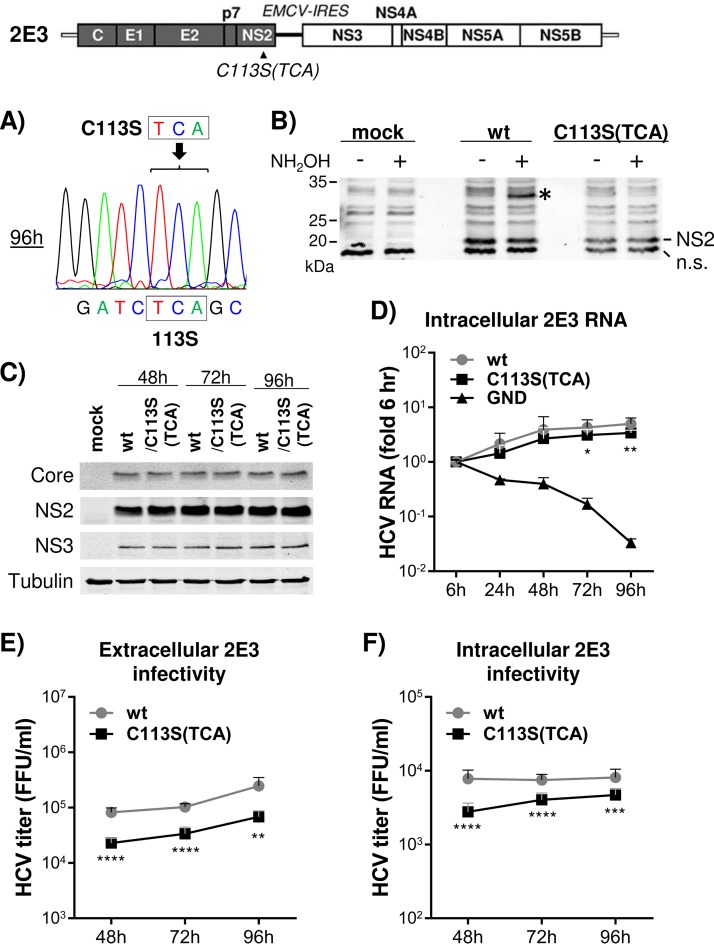
C113S mutation-mediated inhibition of NS2 palmitoylation reduced both intra- and extracellular virus production. (Top) Schematic of 2E3 showing the location of the NS2/C113S(TCA) mutation. (A) Sequencing chromatogram showing maintenance of the NS2/C113S(TCA) mutation until 96 h postelectroporation of mutated 2E3 RNA. (B) Western blot detection of palmitoylated NS2 in cell lysates derived from wt or C113S(TCA) mutated 2E3 RNA replicating cells following APEGS assay. The PEGylated NS2 is indicated by an asterisk. (C) Western blot of HCV Core, NS2, NS3, and tubulin from cell lysates harvested at different time points postelectroporation of wt or NS2/C113S(TCA) mutated 2E3 RNA. (D) Results of quantitative RT (qRT)-PCR to detect HCV RNA present in cell lysates collected at different time points postelectroporation of 2E3 RNA with or without NS2/C113S(TCA) mutation. The graph represents the HCV RNA fold difference relative to the 6-h value. (E and F) Results of extracellular (E) and intracellular (F) viral titration using culture supernatants and cell lysates, respectively, harvested at different time points postelectroporation of wt or NS2/C113S mutated 2E3 RNA. (D, E, and F) The graphs show means with standard deviations from three independent experiments. The asterisks indicate statistically significant differences between paired values based on the Mann-Whitney test. ****, *P* < 0.00005; ***, *P* < 0.0005; **, *P* < 0.005; *, *P* < 0.05.

We determined that an alternative codon (TCA) for the C113S mutation in 2E3 could be maintained up to 96 h into the 2E3 replication period (Fig. 4A). Therefore, we used the NS2/C113S(TCA) mutant in subsequent experiments to assess the functional role of NS2/C113 palmitoylation in HCV replication. The APEGS assay confirmed that the NS2/C113S(TCA) mutation could inhibit NS2 palmitoylation during HCV replication (Fig. 4B). The NS2/C113S(TCA) mutation minimally impacted viral RNA replication, as similar levels of HCV proteins and RNA were detected from wt and NS2/C113S mutant replicating cells (Fig. 4C and D), but reduced both extracellular and intracellular infectious-virus titers (Fig. 4E and F). The slightly lower HCV RNA levels detected from NS2/C113S(TCA) at the 72- and 96-h time points (less than 0. 25-fold) (Fig. 4D) may indicate that the mutation minimally inhibited HCV RNA replication or, more likely, that the phenotype is a secondary effect of reduced viral assembly caused by the mutation. In aggregate, these results suggest that NS2/C113 residue palmitoylation is critical for HCV replication by promoting viral assembly.

### The NS2/C113S(TCA) mutation impaired NS2 and E2 subcellular localization to the DRM.

**FIG 5:**
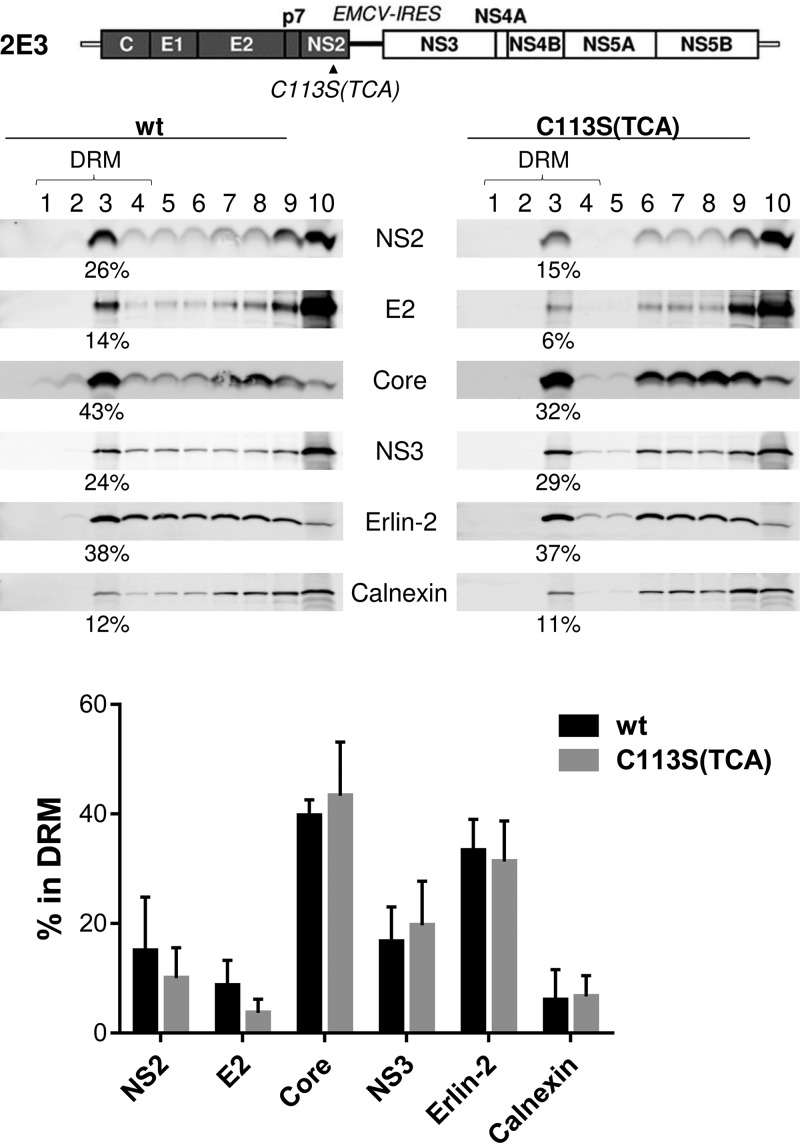
NS2 palmitoylation plays a role in NS2 and E2 localization to the DRM. Shown are the results of a membrane flotation assay performed following cold 1% Triton X-100 treatment of cell lysates harvested from cells electroporated with wt or NS2/C113S(TCA) mutated 2E3 RNA at 72 h postelectroporation (see Materials and Methods for details). The relative percentage of each protein in the DRM fractions was calculated by dividing the levels of protein detectable from the top 4 fractions by those from all fractions. The graph at the bottom shows the mean percentage, with standard deviation, of each protein detected in the DRM fractions from three independent experiments.

Next, we asked whether NS2 palmitoylation at the C113 residue is involved in NS2 localization to the DRM during infectious-HCV replication (21). To determine this, we performed membrane flotation analysis of cold-1% Triton X-100-treated cell lysates derived from 2E3/wt or NS2/C113S(TCA) mutant replicating cells, followed by sampling of 10 different fractions from the top of the gradient, which corresponds to the lowest density. The relative percentage of each protein in the DRM fractions was calculated by dividing the levels of protein detectable from the top 4 fractions by those from all the fractions. As expected, a large portion (∼32% on average among three biological replicates) of ER lipid raft-associated protein 2 (Erlin-2, a marker of the ER-DRM) was localized to the DRM fractions, while a relatively small portion (∼6% on average) of calnexin (a non-DRM ER protein) was found in the DRM fractions (Fig. 5). We also detected >40% of Core and >18% of NS3, on average, in the DRM fractions. The effect of the NS2/C113S mutation on the DRM localization of the above-described host and viral proteins was minimal (Fig. 5, bottom). Under the same experimental conditions, despite the experiment-to-experiment variations of the absolute values of the percentages of proteins detectable in the DRM fractions, the C113S(TCA) mutation consistently reduced NS2 and E2 localization to the DRM by >1.5 fold and >2 fold, on average, respectively (Fig. 5, bottom). These results suggest that NS2/C113 palmitoylation is likely involved in the DRM localization of NS2 and E2. While NS2-dependent E2 localization to the virus assembly sites located at the DRM is well established, the underlying mechanism has been unclear (17–21, 36). Based on our data, we speculate that NS2/C113 palmitoylation regulates NS2 localization to the DRM, which in turn enhances E2 localization to the virus assembly sites located at the DRM.

### NS2/C113S(TCA) mutation reduced NS2 colocalization with Core and Erlin-2.

**FIG 6:**
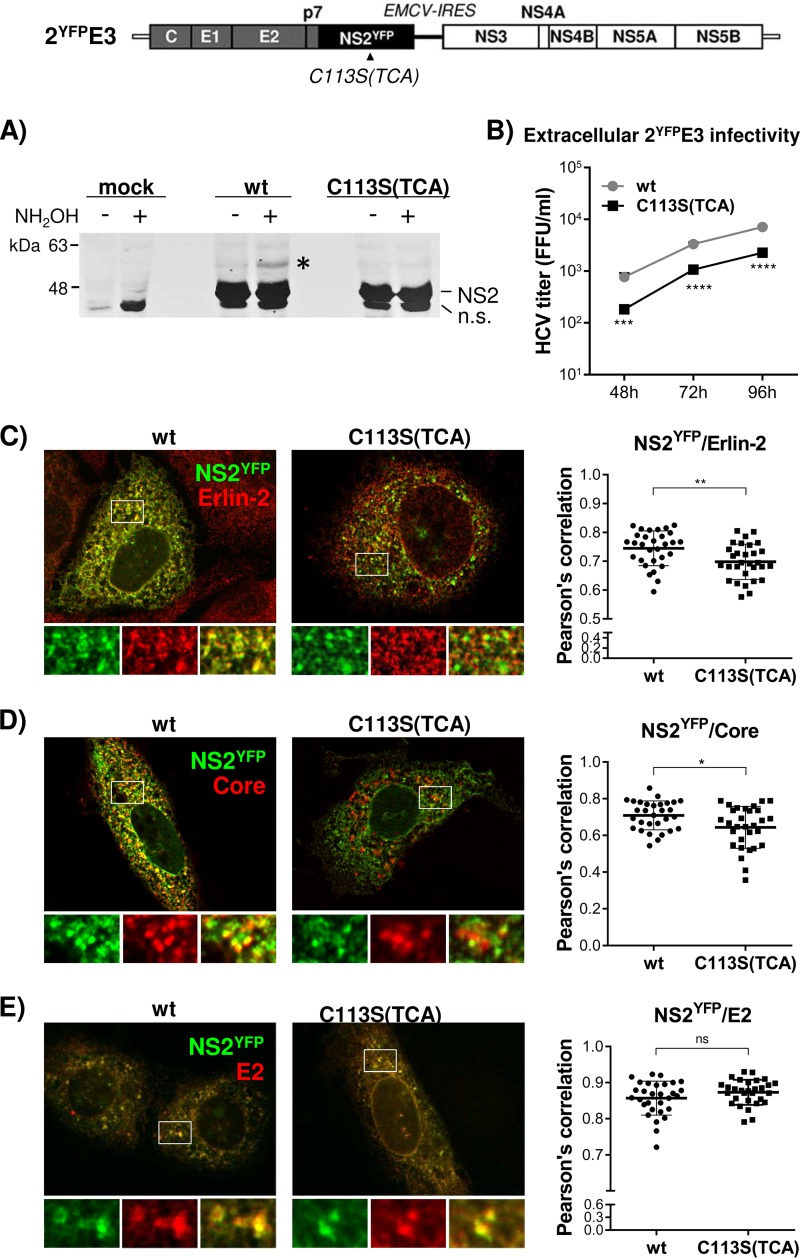
Impaired NS2 palmitoylation reduced the colocalization of NS2^YFP^ with Erlin-2 and Core but not with E2. (Top) Schematic of 2^YFP^E3, which has an organization identical to that of 2E3 but with NS2^YFP^ replacing NS2. (A) Western blot detection of palmitoylated NS2 in cell lysates derived from wt or C113S(TCA) mutated 2^YFP^E3 RNA replicating cells following APEGS assay. The PEGylated NS2^YFP^ is indicated by an asterisk. n.s., nonspecific bands. (B) Extracellular HCV titer determined using culture supernatants collected at different time points postelectroporation of wt or NS2^YFP^/C113S(TCA) mutated 2^YFP^E3 RNA. (C to E) Confocal imaging (left) and Pearson’s correlation coefficient analysis (right) of 30 different images to detect the colocalization of NS2^YFP^ with Erlin-2 (C), Core (D), and E2 (E) in cells at day 3 postelectroporation of wt or NS2^YFP^/C113S(TCA) mutated 2^YFP^E3 RNA. The asterisks indicate statistically significant differences between paired values based on an unpaired Student *t* test with Welch’s correction. *, *P* < 0.05; ** *P* < 0.005. A difference with a *P* value of >0.05 was considered not significant (ns).

**FIG 7:**
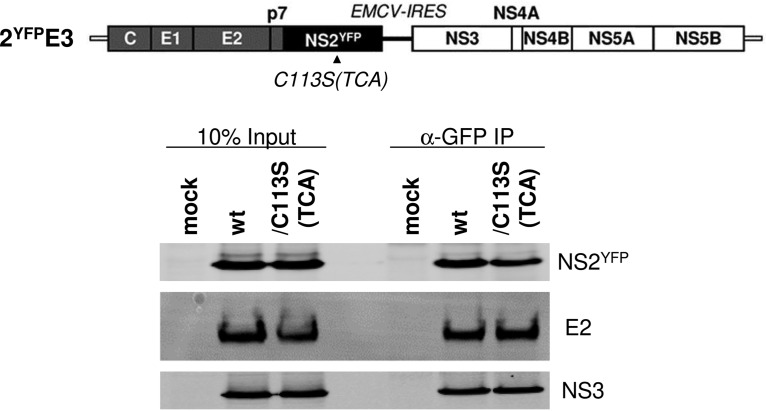
NS2/C113 mutation has no effect on NS2^YFP^-E2 interaction. The interaction of NS2^YFP^ with E2 and NS3 was determined by NS2^YFP^ pull-down from cell lysates prepared at day 3 post electroporation of 2^YFP^E3 RNA. The data presented are representative of the results of three independent experiments.

Next, we assessed NS2 subcellular localization by performing immunofluorescence analysis. To facilitate the detection of NS2, we generated 2E3 encoding yellow fluorescent protein (YFP)-fused NS2 (2^YFP^E3). We determined that YFP fusion to NS2 did not inhibit its palmitoylation (Fig. 6A)—as evidenced by the appearance of an additional, slow-moving, ∼57-kDa YFP-tagged NS2 (NS2^YFP^) band (∼10 kDa higher than the expected molecular weight of NS2^YFP^ at ∼47 kDa) following APGES assay—and permitted infectious-virus production, albeit at a reduced level (Fig. 6B). After confirming that the NS2/C113S(TCA) mutation inhibited NS2^YFP^ palmitoylation, based on the lack of appearance of the ∼57-kDa NS2^YFP^ band following APGES assay of the C113(TCA) mutant (Fig. 6A), and virus production from 2^YFP^E3, as expected (Fig. 6B), we performed confocal imaging analysis to determine NS2 subcellular localization. As shown in Fig. 6C, the NS2/C113S(TCA) mutation significantly reduced NS2^YFP^ and Erlin-2 colocalization, as determined by measuring the Pearson’s correlation coefficient (0.7448 on average for the wt versus 0.6978 on average for the mutant), calculated by using 30 different images derived from three independent experiments. Based on the data shown in Fig. 5, we speculate that NS2/C113S mutation-mediated reduction in NS2 localization to the DRM may be responsible for the decreased colocalization efficiency of NS2^YFP^/C113S and Erlin-2. The NS2/C113S(TCA) mutation also significantly reduced NS2^YFP^ colocalization with the Core puncta, which represent virus assembly sites (37, 38) (Fig. 6D) (Pearson’s correlation coefficient value of 0.7090 on average for the wt versus 0.6437 on average for the mutant). However, the mutation affected neither the colocalization between NS2^YFP^ and E2 (Fig. 6E) nor the coimmunoprecipitation efficiency between the two proteins (Fig. 7). The NS2/C113S mutation also had no effect on NS2 and NS3 interaction, effectively eliminating the possibility of reduced interaction between NS2 and NS3 as a mechanism for decreased NS2 localization to the DRM (Fig. 7). In addition to the lack of a detectable impact of the NS2/C113S mutation on NS2-mediated protein interactions shown above, the mutation also did not alter NS2 mobility when subjected to SDS-PAGE under nonreduced conditions (data not shown). These data imply that the NS2/C113S mutation has minimal impact on NS2 folding (39). In aggregate, these results suggest that NS2 palmitoylation is involved in NS2 and E2 localization to the virus assembly sites located at the ER-DRM without affecting its interaction with E2 and NS3.

## DISCUSSION

NS2 plays a key role in HCV replication by regulating two different steps of the replication. First, as a cysteine protease responsible for autoprocessing at the NS2 and NS3 junction site, with the help of the NS3 cofactor (12, 13), NS2 determines the fate of viral RNA replication, since lack of NS2-NS3 cleavage was shown to impair HCV RNA replication (15). Second, by mediating envelope protein recruitment to the virus assembly sites located at the ER-DRM, NS2 promotes infectious-HCV particle assembly (17–21). Our study provided evidence indicating that NS2/C113 residue palmitoylation is involved in these dual functions of NS2 during HCV replication by enhancing NS2-NS3 autocleavage and promoting NS2 and E2 localization to the DRM.

**FIG 8:**
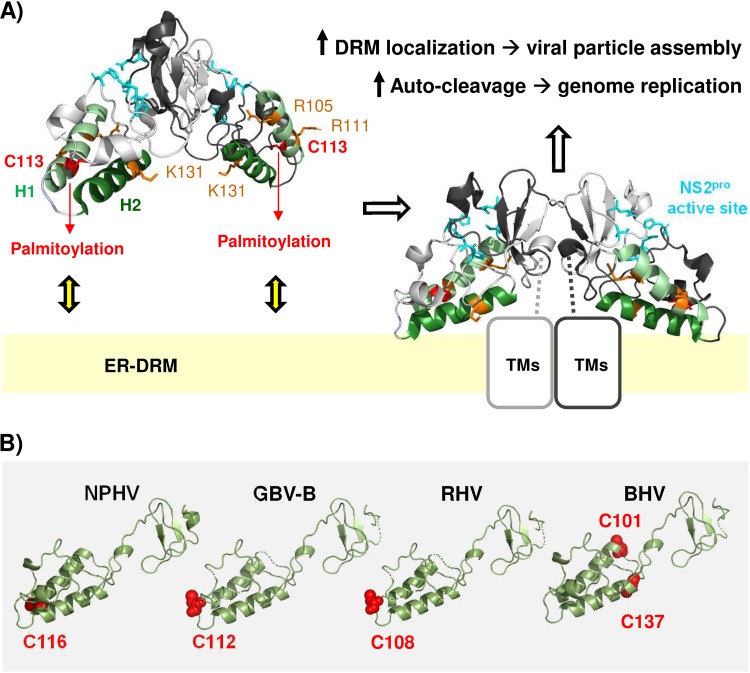
Model for the functional role of NS2/C113 palmitoylation. (A) NS2/C113 palmitoylation in helix 1 (H1), potentially assisted by positively charged residues in H1 and H2, promotes the membrane association of H1 and H2, properly orienting the composite catalytic domains of dimeric NS2 toward the cytoplasm for efficient autocleavage at the NS2-NS3 junction site and facilitating the association of N-terminal transmembrane domains of NS2 (TMs) with the ER-DRM, thereby enhancing HCV particle assembly (see Discussion for details). The structural model was based on the crystal structure of the gt1a HCV NS2 protease domain (RCSB Protein Data Bank [PDB] entry 2HD0) (11; http://www.rcsb.org). (B) We used the PyMOL Molecular Graphics System v2.0.6 (DeLano Scientific, San Carlos, CA, USA) (http://www.pymol.org) to create the protein structure images. The protein sequences were retrieved from the NCBI GenBank database (51): NPHV H3-011 (JQ434008), GBV-B (AY243572), RHV NLR07-oct70 (KC411784), and BHV PDB-452 (KC796090). An alignment of sequences was computed by MUSCLE (52) and improved by minor manual modifications in SeaView (53) according to the alignment published by Boukadida et al. (12). Homology-derived three-dimensional (3D) structure models for New World primate hepacivirus (GBV-B) and equine (NPHV), rodent (RHV), and bat hepaciviruses (BHV) were constructed using the WHAT IF modeling server (54) with sequence structure alignments based on PDB entry 2HD0 (chain A) from the RCSB PDB (http://www.rcsb.org) (11), comprising the crystal structure of the catalytic domain of the hepatitis C virus NS2-3 protease from genotype 1a (isolate H).

Our data showing the NS2/C113S mutation-mediated inhibition of NS2-NS3 autoprocessing in cells are consistent with a previous report by Tedbury and Harris, who demonstrated the NS2/C113A mutation-mediated inhibition of purified NS2-NS3 autoprocessing (35). Since we used gt1a NS2-NS3 and the above-mentioned study used gt1b NS2-NS3, these results reinforce the important role of the NS2/C113 residue in NS2-NS3 autoprocessing. The C-terminal domain of NS2 functions as a dimeric cysteine protease with two composite active sites (11, 12). According to the crystal structure of the C-terminal domain of NS2 encompassing residues 94 to 217 (NS2_94–217_), reported by Lorenz et al. (11), residue C113 is located at H1, which is the first of the two alpha helices (H1 and H2) preceding the composite protease active sites (Fig. 8A). Palmitoylation would increase the hydrophobicity of the NS2/C113 residue, consequently attracting H1 and H2 to the ER membranes (40) (Fig. 8A). Then, two conserved arginine residues in H1 (R105 and R111) and a single lysine or arginine residue in H2 (K131 in gt1a, gt4, and gt6 or R134 in gt2a, gt3, and gt5), along with numerous hydrophobic residues within H1 and H2, would provide a favorable environment for H1 and H2 to interact with the membranes (11, 41) (Fig. 8A). Alternatively, it is equally possible that interaction of the H1 and/or H2 domain with the membrane surface promoted NS2/C113 palmitoylation by bringing the residue close to the membrane-embedded palmitoyl acyltransferase (PAT) (42, 43). Supporting the latter possibility, Lange et al. showed that H2 alone could confer membrane association of a green fluorescent protein (GFP) reporter and that a single K131A mutation in H2 within the NS2_96–217_ domain fused to GFP could alter the membrane association phenotype of the reporter fragment (41). Interestingly, the above-mentioned study also showed that NS2-NS3 autoprocessing was inhibited by the K131A mutation in NS2 (41). In other words, the NS2/K131A mutation altered both the membrane association of the H2 domain and the NS2-NS3 cleavage. NS2/C113S likely had reduced the H1 domain’s membrane association by preventing NS2 palmitoylation in the H1 domain. Since the NS2/C113S mutation reduced NS2-NS3 autoprocessing, together with the phenotypes of the NS2/K131A mutation described above, these data suggest that the membrane association of the H1 and H2 domains in NS2 modulates NS2-NS3 self-cleavage efficiency. Interaction of H1 and H2 domains with the membranes should stabilize the proper orientation and/or dimeric interaction of the downstream cysteine protease domain of NS2 to efficiently process NS2-NS3 precursor (Fig. 8A).

NS2 is an integral membrane protein including at least three transmembrane domains at its N terminus (17, 44). Palmitoylation of integral membrane proteins was dependent on the accessibility of the PAT to cysteines and occurred on cysteines located in the cytoplasmic region less than 30 amino acids away from the transmembrane border or situated up to 8 Å into the inner leaflet of the membrane (42, 43). NS2/C113 in the cytoplasmic domain of NS2 is just 18 amino acids away from the last residue of the third transmembrane alpha helix of NS2 (17) and is situated in a preferred palmitoylation sequence motif (Fig. 1A), providing an ideal context for palmitoylation. A recent study by Boukadida et al. showed that NS2 protease domains from HCV and other, related hepaciviruses share composite active-site-mediated NS2-NS3 autocleavage (12). The same study presented an HCV NS2 structure-based homology model suggesting the homologous three-dimensional dimeric folds of the NS2 protease domain, as well as H1 and H2 domains, among different hepaciviruses (12). Curiously, according to our analysis, most of the hepaciviruses analyzed in the above-mentioned study encode a cysteine residue at a location exactly matching or near the HCV NS2/C113S residue, including C116 of nonprimate hepacivirus (NPHV) or equine hepacivirus, C112 of GB virus B (GBV-B), C108 of RHV, and C101 and C137 of bat hepacivirus (BHV) (see Fig. 8B). Since these cysteine residue locations fall into a PAT-accessible area, it is possible that palmitoylation of a cysteine residue in H1 (or H1 and H2 in the case of BHV) may be a conserved feature shared between HCV and the other hepaciviruses. However, this aspect needs to be confirmed in the future.

Protein S-palmitoylation is the only reversible protein lipidation that allows dynamic regulation of the function and subcellular localization of proteins (45). The thioesterification of a 16-carbon saturated fatty acid (palmitate) to an internal cysteine residue is catalyzed in mammals by a family of 23 PATs, which share a conserved aspartate-histidine-histidine-cysteine (DHHC) motif (46). Here, we determined that H77 NS2 localization to the DRM is dependent on its C113 residue palmitoylation (Fig. 5 and 6C). This result is consistent with the role of protein palmitoylation as a major regulator of transmembrane protein lipid raft (DRM) affinity (24). HCV p7 may be involved in the regulation of NS2 palmitoylation, since NS2 localization to the DRM was shown to be dependent on functional p7 (21). Since p7 itself did not significantly localize to the DRM (21), p7’s role in NS2 recruitment to the DRM likely involves an indirect mechanism, such as mediating the interaction between PAT and NS2. While it is beyond the scope of the current investigation, defining the mechanism of action for p7-dependent NS2 localization to the DRM, potentially by enhancing NS2 palmitoylation, will allow us to better define the interplay between viral proteins to fine tune the HCV life cycle.

In conclusion, our data provide evidence for the dual functional roles of gt1a H77 NS2 palmitoylation: inducing HCV RNA replication by promoting the cleavage of NS2-NS3 precursor and enhancing virus assembly by mediating NS2-dependent E2 localization to the virus assembly sites at the ER-DRM. At the moment, it remains unclear whether NS2 palmitoylation is an HCV genotype-dependent or pangenotypic phenotype. However, complete conservation of the NS2/C113 residue among different HCV genotypes (Fig. 1B), as well as the presence of the equivalent cysteine(s) at the structurally homologous area in NS2 from diverse members of other hepaciviruses (Fig. 8B), suggests that HCV NS2/C113 or an equivalent cysteine residue(s) in other hepaciviruses plays an evolutionarily conserved role in the hepacivirus life cycle, at least for some members of the genus, in a palmitoylation-dependent manner. Proving the functionally equivalent roles of NS2 palmitoylation in other mammalian hepaciviruses in future studies will likely pave the way to comprehensively define the role of NS2 palmitoylation in the hepacivirus life cycle by using surrogate immunocompetent animal models of HCV infection (5, 47).

## MATERIALS AND METHODS

### Cell culture.

HEK293T cells and clonal derivatives of Huh7 cells, including Huh7.5 (kindly provided by Charles Rice, Rockefeller University [48]) and FT3-7 (32) cells, were cultured in Dulbecco’s modified Eagle’s medium (DMEM) containing 10% fetal bovine serum (Invitrogen, Carlsbad, CA) at 37°C under a humidified atmosphere with 5% CO_2_.

### Plasmids.

The cDNAs encoding HCV NS2 and NS234 with an N-terminal Flag (DYKDDDDK) epitope tag were amplified from HCV genotype 1a (H77S) (49) and cloned into a pcDNA6/V5-His vector (Invitrogen, Carlsbad, CA) to generate p^Flag^NS2 and p^Flag^NS2-NS3-NS4A. A mutation replacing cysteine at NS2 residue 113 with serine (NS2/C113S) was introduced by performing site-directed mutagenesis with the primer set forward (5′-CAAGGCCTTCTCCGGATCTCGGCGCTAGCGCGGAAGATAG-3′) and reverse (5′-CTATCTTCCGCGCTAGCGCCGAGATCCGGAGAAGGCCTTG-3′) or, alternatively, with the primer set forward (5′-CAAGGCCTTCTCCGGATCTCAGCGCTAGCGCGG-3′) and reverse (5′-CCGCGCTAGCGCCGCTGAGATCCGGAGAAGGCCTTG-3′) in the case of NS2/C113S(TCA). Infectious-HCV clonal constructs HJ3-5/2E3 and HJ3-5/2^YFP^E3 were generated by introducing the EMCV IRES between NS2 and NS3 in HJ3-5 (31) and HJ3-5/NS2^YFP^ (20). To do this, a PmeI restriction site was introduced between the NS2 and NS3 regions of HJ3-5 or HJ3-5/NS2^YFP^. Then, overlapping PCR was carried out to generate an EMCV-IRES-NS3 fragment with an N-terminal PmeI site. This fragment was digested with PmeI and AvrII (located in JFH-1 NS3) restriction enzymes and ligated to PmeI/AvrII vector fragments derived from pHJ3-5/PmeI and pHJ3-5/NS2^YFP^/PmeI to make pHJ3-5/2E3 and pHJ3-5/2^YFP^E3. NS2/C113S and NS2/C113S(TCA) mutations were introduced into these plasmids as described above. The sequences of the regions manipulated within each plasmid were verified by DNA sequencing.

### DNA transfection.

p^Flag^NS2 and p^Flag^NS234A were transfected into HEK293T cells using TransIT-LT1 reagent (Mirus Bio LLC, Madison, WI, USA) according to the protocol recommended by the manufacturer.

### Cell fractionations.

Cell fractionations into detergent-soluble and -insoluble fractions were performed according to methods described previously (21, 30). Briefly, cells grown on 12-well plates were washed twice with phosphate-buffered saline (PBS) and lysed in lysis buffer (1% Triton X-100 and 10% [vol/vol] glycerol in PBS) supplemented with protease inhibitor cocktail (GenDepot, Barker, TX). After incubation on ice for 15 min, a detergent-soluble fraction (supernatant) was collected by centrifugation at 500 × *g* for 5 min at 4°C. The detergent-insoluble fraction (pellet) was washed twice in lysis buffer. The proteins in the detergent-soluble and -insoluble fractions were then analyzed by Western blotting.

### Quantitative real-time RT-PCR assay.

Intracellular HCV RNA was isolated using an RNeasy RNA isolation kit (Qiagen, Valencia, CA). To quantitate the level of HCV RNA, a real-time reverse transcription (RT)-PCR assay was performed using a QuantiNova Probe RT-PCR kit (Qiagen, Valencia, CA) and a CFX96 real-time system (Bio-Rad, Hercules, CA) with custom-designed primer-probe sets (a forward primer, HCV84FP, 5′-GCCATGGCGTTAGTATGAGTGT-3′; a reverse primer, HCV303RP, 5′-CGCCCTATCAGGCAGTACCACAA-3′; and a probe, HCV146BHQ, 6-carboxyfluorescein [FAM]-TCTGCGGAACCGGTGAGTACACC-DBH1 [dual-labeled probe Black Hole Quencher 1]), as described in detail previously (50).

### Western blot analysis.

Cell lysates were prepared in lysis buffer (50 mM Tris-HCl, 150 mM NaCl, 1% Triton X-100, 0.5% sodium deoxycholate, pH 7.5, and 2 mM EDTA) containing 1× protease and phosphatase inhibitor cocktail mixture (GenDepot, Katy, TX), separated by SDS-PAGE, and transferred onto polyvinylidene difluoride (PVDF) membranes. The membranes were blocked and probed with primary antibodies to Core (1:2,000 dilution of C7-50; Thermo Scientific, Rockford, IL), NS3 (1:2,000 dilution of 9-G2; ViroGen, Watertown, MA), NS2 (polyclonal rabbit anti-NS2 antibody) (20), E2 (1:2,000 dilution of polyclonal goat anti-E2 antibody (Virostat, Inc., Portland, ME), calnexin (1:5,000 dilution of polyclonal rabbit anti-calnexin antibody; Calbiochem), fliotillin-1 (1:2,000 dilution of monoclonal mouse anti-flotillin-1; BD Transduction Laboratories), and tubulin (1:7,000 dilution; EMD Millipore, Billerica, MA). Protein bands were visualized by incubating the membranes with IRDye secondary antibodies (Li-Cor Biosciences, Lincoln, NE), followed by imaging with an Odyssey infrared imaging system (Li-Cor Biosciences, Lincoln, NE).

### *In vitro* HCV RNA transcription and electroporation.

HCV cDNA was linearized with XbaI (NEB, Hitchin, United Kingdom), followed by transcription to RNA using a T7 Megascript kit (Ambion, Austin, TX). HCV RNA was purified using an RNeasy RNA isolation kit (Qiagen, Valencia, CA). For electroporation, 10 μg of RNA was mixed with 5 × 10^6^ FT3-7 cells in a 0.4-cm gap width electroporation cuvette (Bio-Rad, Hercules, CA) and pulsed once at 270 V and 950 μF using a GenePulser system (Bio-Rad, Hercules, CA).

### HCV infectivity assays.

For virus titration, 50-μl aliquots of serial 10-fold dilutions of cell culture supernatant fluids, clarified by low-speed centrifugation, were inoculated onto naive Huh-7.5 cells in a 96-well plate. After 72 h, the cells were fixed with 4% paraformaldehyde (PFA) for 20 min and then stained with anti-Core antibody (Affinity BioReagents; 1:1,000). For intracellular-virus titration, cells were trypsinized, resuspended in 500 μl medium, and lysed by 4 freeze-thaw cycles. Clarified cell lysates were used to inoculate Huh-7.5 cells as described above. HCV infectivity was determined by counting the clusters of Core-immunostained cells (focus), and the titer was expressed as focus-forming units (FFU) per milliliter.

### Confocal microscopy.

Electroporated cells were plated on 8-well chamber slides (BD Biosciences, Bedford, MA) at a density of 1 × 10^4^ cells per well. Two days later, the electroporated cells were fixed in 4% paraformaldehyde. After blocking nonspecific binding by incubating the cells in a PBS solution containing 3.5% bovine serum albumin for 1 h at room temperature, the cells were incubated with primary antibodies for 2 h and then with secondary antibodies for 1 h at room temperature. The slides were examined with an Olympus FluoView FV10i confocal microscope (Olympus America Inc., Waltham, MA). Pearson’s coefficient was obtained by using FV10i-ASW 4.2 viewer software.

### Membrane flotation assay.

Membrane flotation was performed according to methods described previously (21). Briefly, the cells were electroporated with HCV RNA, washed with ice-cold PBS, and then scraped in 0.4 ml of TNE buffer (25 mM Tris-HCl [pH 7.4], 150 mM NaCl, 5 mM EDTA) containing a protease inhibitor cocktail mixture (GenDepot, Barker, TX). The cells were then disrupted by passing them through a needle 50 times and incubated on ice in the absence or presence of 1% Triton X-100 for 30 min. The cell lysates were then mixed with 0.4 ml of 60% iodixanol (Sigma, St. Louis, MO), resulting in a 40% iodixanol concentration. A discontinuous iodixanol gradient (30%, 26%, and 6%) was layered on top of the lysate-iodixanol mixture and ultracentrifuged at 42,000 rpm for 4 h at 4°C in an SW60 rotor. A total of 10 fractions (400 μl each) were collected from the top and precipitated after adding 100 μl of trichloroacetic acid (100% [wt/vol]). The precipitates were washed twice with cold acetone and solubilized with 2× SDS sample buffer before subjecting them to SDS-PAGE and Western blotting.

### APEGS assay.

A previously described protocol was followed to perform the APEGS assay (28, 33). Briefly, cells were lysed with TES buffer (50 mM Tris, pH 7.4, 150 mM NaCl, 5 mM EDTA, 0.5 % TX-100, 0.1 % SDS) and 2× protease inhibitor. The clarified cell lysates were treated with Tris(2-carboxyethyl)phosphine hydrochloride (TCEP HCl) at a final concentration of 10 mM and incubated for 30 min at room temperature. Then, NEM and SDS were added to final concentrations of 10 mM and 0.5% (wt/vol), respectively, followed by overnight incubation at 4°C. The reaction was terminated by adding methanol-chloroform-H_2_O, and the precipitated proteins were dissolved in 60 μl PR buffer (4 % SDS, 50 mM Tris, pH 7.4, 5 mM EDTA). Half of the dissolved-protein solution was mixed with 50 μl of 1 M hydroxylamine (pH 8.0), while the remaining half was mixed with 50 μl of 1 M Tris (pH 8.0), followed by incubation at room temperature for 1 h. Each reaction was stopped by adding methanol-chloroform-H_2_O, and the precipitated pellets were resuspended in 30 μl of PR buffer. Next, the protein solution was mixed with 60 μl of 2 % (wt/vol) mPEG-mal (Sigma, St. Louis, MO) in TES lysis buffer and incubated at 25°C for 2 h. Finally, samples were reprecipitated and analyzed by Western blotting.

### Statistical analyses.

Mann-Whitney tests and unpaired Student's *t* tests with Welch’s correction were performed using GraphPad Prism version 6 software to determine the significance of differences between paired values from at least three independent experiments. A *P* value of less than 0.05 was considered statistically significant.
